# Biotic Elicitors in Adventitious and Hairy Root Cultures: A Review from 2010 to 2022

**DOI:** 10.3390/molecules27165253

**Published:** 2022-08-17

**Authors:** Miguel Angel Alcalde, Edgar Perez-Matas, Ainoa Escrich, Rosa M. Cusido, Javier Palazon, Mercedes Bonfill

**Affiliations:** 1 Laboratorio de Fisiologia Vegetal, Facultad de Farmacia, Universitat de Barcelona, Avda. Joan XXIII 27-31, 08028 Barcelona, Spain; 2 Department of Medicine and Life Sciences, Universitat Pompeu Fabra, 08003 Barcelona, Spain

**Keywords:** elicitor, secondary metabolites, hairy root, adventitious root, production, biotic elicitor, phytochemical

## Abstract

One of the aims of plant in vitro culture is to produce secondary plant metabolites using plant cells and organ cultures, such as cell suspensions, adventitious, and hairy roots (among others). In cases where the biosynthesis of a compound in the plant is restricted to a specific organ, unorganized systems, such as plant cell cultures, are sometimes unsuitable for biosynthesis. Then, its production is based on the establishment of organ cultures such as roots or aerial shoots. To increase the production in these biotechnological systems, elicitors have been used for years as a useful tool since they activate secondary biosynthetic pathways that control the flow of carbon to obtain different plant compounds. One important biotechnological system for the production of plant secondary metabolites or phytochemicals is root culture. Plant roots have a very active metabolism and can biosynthesize a large number of secondary compounds in an exclusive way. Some of these compounds, such as tropane alkaloids, ajmalicine, ginsenosides, etc., can also be biosynthesized in undifferentiated systems, such as cell cultures. In some cases, cell differentiation and organ formation is necessary to produce the bioactive compounds. This review analyses the biotic elicitors most frequently used in adventitious and hairy root cultures from 2010 to 2022, focusing on the plant species, the target secondary metabolite, the elicitor and its concentration, and the yield/productivity of the target compounds obtained. With this overview, it may be easier to work with elicitors in in vitro root cultures and help understand why some are more effective than others.

## 1. Introduction

Secondary metabolite production is connected with the differentiation of the plant, and means that secondary pathways are activated in the plant as a whole. Unlike callus and cell suspension cultures, roots are organs that maintain the same structure as in the plant, so its differentiation allows a directed activation of the secondary metabolism. In fact, cell differentiation and organ formation are, in some cases, necessary for producing the bioactive compounds. For this reason, in the middle of the last century, plant biotechnologists began to work in root cultures. The problem of this type of cultures was the low growth rate and in some cases the addition of indol-acetic acid (IAA) to promote growth caused low production of the bioactive compounds.

During this period, hairy root syndrome was discovered in plants in nature, which is induced by *Agrobacterium rhizogenes* [1]. *A. rhizogenes* is a bacterium that infects plants in nature transferring a part of its plasmid DNA to the plant cell, the transferred DNA (T-DNA). The genetically transformed cells develop roots called hairy roots or transformed roots. Hairy root cultures show a very high biomass production and a metabolic profile similar to the root of the whole plant.

The genes of the T-DNA which are responsible for the root development [2] are the *rol* genes. Among the *rol* genes, the most important are *rol A* gene which increases the sensitivity of the transformed cell to auxin [3], the *rol B* gene which has a role in signal transduction related to auxin sensitivity [4] as confirmed by its tyrosine phosphatase activity [5] (this *rol* gene is crucial for hairy root initiation and elongation, and especially, in meristem formation [6]), and the *rol C* gene which hydrolizes the conjugates of cytokinins [3].

This review focuses on one of the strategies to improve the secondary metabolite production in adventitious and hairy root cultures, that is, the addition of biotic elicitors to the culture medium. It screens the biotic elicitors most utilized in root in vitro cultures in the last decade and analyzes the production of the secondary metabolites in relation to the type of elicitor, its concentration and the plant species. A comparison between hairy roots and adventitious roots is made and a discussion about the improvement of yield/productivity of the target secondary metabolite with different biotic elicitors is shown.

In addition, Excel 2010, GraphPad Prism 8 and Tableau 2020.1 were used to organize and analyze data on the maximum production value, range and the most common studied elicitor by group of metabolites, plant family and origin of the research group.

All information about the biotic elicitors used during the last decade were collected from research articles and reviews from 2010 until 2022. The electronic databases employed to obtain relevant information include Web of Science (accessed on 1 April 2022), Scopus (accessed on 1 April 2022) and PubMed (accessed on 1 April 2022).

The analyzed data includes the biotic elicitors most used in hairy root cultures from 2010 to 2022. The data, 87 studies, were organized and analyzed using Microsoft Excel 2016 (Microsoft Corporation, Redmond, WC, USA, 2016), GraphPad Prism 8 (GraphPad Prism Inc., San Diego, CA, USA, 2019) and Tableau 2020.1 (Salesforce, San Francisco, CA, USA, 2019).

## 2. Root In Vitro Cultures

### 2.1. Hairy Roots

After infection of the explant (leave, stems, rhizome, etc.) with *A. rhizogenes*, the obtained hairy roots are cultured in a solid medium for growth and confirmation of their transformed nature.

The confirmed transformed roots are then transferred to liquid medium to optimize their growth and production. The hairy root methodology is based on optimized protocols for the genetic transformation of all species that have been investigated, which include the isolation and selection of the most productive root lines, optimization of the culture conditions by assaying several basic media, plant growth regulators (PGRs), sugar supplements, addition of elicitors, precursors, etc., to improve the production, and finally the scale-up to bioreactors [7]. At this level, the scaling-up process is very difficult since bioreactors are generally designed for the culture of microorganisms and need to be modified to adapt the transformed roots to the new culture conditions.

Some examples of hairy root cultures are transformed root cultures for the production of ajmalicine in *Catharanthus roseus* [8], ginsenosides in *Panax ginseng* [9], tropane alkaloids in *Datura metel* [10], *Duboisia* sp. [11], *Brugmansia candida* [12] and *Hyoscyamus niger* [13], withanolides in *Withania coagulans* [14], taxol in *Taxus* spp. [15] and hairy roots of *Linum album* for the production of podophyllotoxin and methoxypodophyllotoxin [16]. The advantages of the hairy root cultures are the following: there is fast growth in culture media without PGRs; they are economically feasible since high-cost media constituents are not needed; the product is obtained without chemical alterations; and the level of the production is predictable, genetic stability over long culture periods and large-scale cultivation without loss of biosynthetic capacity [17].

One strategy to work during long periods with the same roots (known as a continuous system) and to increase production involves stimulating the release of the target compound to the culture medium. Root cultures frequently accumulate the bioactive compounds inside the cells in vacuoles, so it is necessary to harvest the biomass of the culture in order to extract the bioactive compounds [17]. Extraction from dry plant matter is a difficult process due to the high quantity of waxes and pigments. Permeabilizing agents, as dimethyl sulfoxide, can release the phytochemicals caught in the root cells to the culture medium, thus facilitating the final extraction process [18].

As the *Agrobacterium* infection is cell by cell, each root that appears from an inoculated explant should be isolated and treated as an individual hairy root line. In this way, one can obtain several lines of hairy roots that usually have different morphologies. A study with *Panax ginseng* hairy roots, showed three types of morphologies from the same type explant: 50% of lines with a typical hairy root morphology, 35% with a thick morphology, and 15% with a thin morphology. The behavior of these roots was different in growth and ginsenoside production. However, the metabolic profile was not altered, and interestingly, the same elicitor increased the production in all types of roots [19].

### 2.2. Adventitious Roots

Adventitious roots constitute another in vitro culture system to produce secondary metabolites [20]. Although they normally have a low growth rate, they can be easily cultured in vitro with the addition of low concentrations of auxin [21], using a similar methodology to that of hairy roots, with the advantage that the infection process with *Agrobacterium* is avoided. Being a differentiated culture system, adventitious roots also possess genetic and biosynthetic stability [22] and maintain the biosynthetic profile of the target compounds as in hairy roots [16].

## 3. Use of Elicitors to Increase the Production

Elicitation is one of the most effective techniques currently used for improving the biotechnological production of plant secondary metabolites (PSMs). The use of this biotechnological tool showed good results in different culture systems and several plant species [23,24].

Elicitors are compounds that stimulate any type of plant defense, promoting secondary metabolism to protect the cell and the whole plant [25,26,27,28] since they can trigger the expression of key genes in the secondary biosynthetic pathways.

Also, it has been defined that an elicitor is a substance that, when introduced in small concentrations to a living cell system, initiates or improves the biosynthesis of specific compounds [29]; elicitation is a process of induced or enhanced plant biosynthesis of secondary metabolites due to the addition of trace amounts of elicitors [30]. Depending on their origin, elicitors can be classified into abiotic (such as metal ions, inorganic compounds) and biotic (including polysaccharides derived from plant cell walls, micro-organisms and glycoproteins) [30,31].

### 3.1. Biotic Elicitors

Biotic elicitors have a biological origin, and include substances derived from pathogens (exogenous elicitors) and compounds produced by plants after the action of the pathogen (endogenous elicitors) [32]. Exogenous biotic elicitors include compounds released by microorganisms and other pathogens, or formed by the action of plant enzymes on microbial cell walls, such as microbial enzymes, fungal and bacterial lysates, yeast extracts and polysaccharides from microorganisms’ cell walls (e.g., chitin and glucanes) [33].

Endogenous biotic elicitors include polysaccharides arising from pathogen degradation of the plant cell wall, intracellular proteins and small molecules synthesized by plant cells in response to different types of stress or pathogen attack, including PGRs such as methyl jasmonate (MeJA) or salicylic acid (SA) [28].

The main biotic elicitors used in this period are: Acetylsalicylic acid (ASA), Chitosan (CS), Coronatine (COR), Jasmonic acid (JA), MeJA, Pectin, SA and Yeast extact (YE).

#### 3.1.1. Acetylsalicylic Acid and Salicylic Acid

Salicylic acid (SA) is known to induce systemic acquired resistance to many pathogens [34]. Among the wide range of defense responses, it is included the production of PSM. However, SA is not a global elicitor, and induces only certain classes of secondary metabolites [35]. A derivative of SA, acetylsalicylic acid (ASA), has been used as elicitor due to its similar chemical structure [36].

#### 3.1.2. Chitosan

Chitosan (CS) is a carbohydrate generated from chitin, a cell wall component of fungi and yeast, by partial deacetylation under alkaline conditions or enzymatic hydrolysis by chitin deacetylases [37].

#### 3.1.3. Coronatine

Coronatine (COR), a polyketide phytotoxin produced by microbes, is a non-host specific toxin that causes diffuse chlorosis in a wide variety of plant species [38]. The mechanism underlying the effects of COR is based on an ability to mimic a bioactive jasmonic acid conjugate (JA-Ile) and thus target the JA-receptor. COR directly harnesses JA-signal transduction proteins to hijack hormone signaling. In this way, it can suppress SA-dependent defenses, impede stomatal closure to provide pathogens with access to plant leaves, and suppression of plant cell wall defense through disturbance of secondary metabolism [39].

#### 3.1.4. Jasmonic Acid and Methyl Jasmonate

Jasmonates (JAs), particularly methyl jasmonate (MeJA) are plant-specific molecules whose biosynthesis is induced by pathogen attack and wounding [35]. When exogenously applied to plant cell cultures, JAs stimulate secondary biosynthetic pathways and increase the production of secondary metabolites belonging to the three main groups: terpenoids, alkaloids, and phenylpropanoids [28]. An interesting review of jasmonic acid (JA) and MeJA has been published by Nabi et al. [40].

MeJA is the elicitor most used to enhance secondary metabolite production in root in vitro cultures, and specifically, at the concentration 100 μM. Different PSMs, belonging to the main three groups (terpenes, alkaloids and phenols), respond to the elicitation of MeJA increasing its production [41].

#### 3.1.5. Pectin

Pectin, an essential branched polysaccharide, is a major component of primary cell walls of all land plants and encompasses a range of galacturonic acid-rich polysaccharides. Three major pectic polysaccharides (homogalacturonan, rhamnogalacturonan-I and rhamnogalacturonan-II) are thought to occur in all primary cell walls. The highest concentrations of pectin are found in the middle lamella of the cell wall, decreasing gradually through the primary wall toward the plasma membrane [42].

#### 3.1.6. Yeast Extract

The knowledge of the composition and mechanism of action of yeast extracts (YEs) on PSM production is limited and yet full entirely in the empirical approach. However, they are widely used as elicitors to activate plant secondary biosynthetic pathways.

## 4. The Main Biotic Elicitors Used in Hairy Roots from 2010 to 2022

Table 1 summarizes the data on elicitation of hairy root cultures from 2010 to 2022.

Several examples are outlined here, grouping different studies according to the type of secondary metabolite. When a biotic elicitor is not mentioned in a group of secondary metabolites, it is due to the fact that no references have been found.

### 4.1. Alkaloids

#### 4.1.1. Acetylsalicylic Acid and Salicylic Acid

(A) The yield of the tropane alkaloids hyoscyamine and scopolamine increased after elicitation with 1000 μM ASA in hairy root cultures of *Anisodus luridus*, reaching 57.2 and 14.7 mg/g dry weight (DW), respectively. One thousand μM ASA also strongly induced the release of scopolamine to the culture medium, resulting in a content of 1.02 mg/mL, which is 6.2-fold higher compared to the control [52].

(B) The highest accumulation of hyoscyamine and scopolamine in *Hyoscyamus reticulatus* L. hairy roots elicited with ASA (1.6- and 3.5-fold higher than in the control, respectively) was obtained at 24 h of exposure to 100 μM ASA. In correlation with these results, semi-quantitative RT-PCR analysis revealed an increased expression of the hyoscyamine-6-beta-hydroxylase (*h6h*) gene, involved in the last biosynthetic step of these tropane alkaloids [56].

(C) A study on transgenic hairy root lines of *Datura stramonium*, *D. innoxia* and *D. tatula* revealed that the optimal elicitation conditions for the highest production of hyoscyamine was with 0.1 μM ASA, yielding up to 7.94 ± 0.14 mg/g DW [48].

(D) Elicitation of *Isatis tinctoria* L. hairy roots with 142.61 mM SA increased the alkaloid content 5.89-fold compared with the control hairy roots. In this study, SA was more effective than ASA and MeJA. The best results for all these elicitors were achieved within the concentration range of 100–200 mM [43].

(E) Diploid and tetraploid hairy roots of *Datura stramonium* were elicited with 100 mM SA and 100 mM ASA. Both elicitors increased hyoscyamine production, although the best treatment for both types of roots was 100 mM SA, which increased the hyoscyamine content to 7.697 mg/g DW in the diploid line and 12.315 mg/g DW in the tetraploid line, an improvement of 190% and 126%, respectively. A synergistic effect of polyploidization and elicitation was observed [49].

#### 4.1.2. Chitosan

The production of trigonelline, an alkaloid synthesized by fenugreek hairy roots (*Trigonella foenum-graecum*), was studied using different concentrations of MeJA (0, 25, 50, 100 and 200 µM) and CS (0, 50, 100, 150, and 200 mg/L). The highest content of trigonelline (37.3 mM/g DW) was obtained with an elicitation treatment of 150 mg/L CS. It was demonstrated that hairy root cultures, in addition to being fast-growing, have rates of secondary metabolite production equal to or greater than the intact plant [55].

#### 4.1.3. Coronatine

Fattahi et al. [57] studied the effect of the new elicitors methyl-β-cyclodextrins (β-CD) and COR on tropane alkaloid biosynthesis in *Atropa acuminata* and *A. belladonna* hairy root cultures. Selected hairy root lines of both species were elicited with 50 mM β-CD, 0.5 μM COR or 50 mM β-CD + 0.5 μM COR on day 14 of culture. In *A. belladonna* hairy roots all the elicitor treatments had a negative effect on both hyoscyamine and scopolamine production, whereas in those of *A. acuminata*, COR elicitation increased the scopolamine content 10-fold compared to the control (10.95 mg/g DW).

#### 4.1.4. Jasmonic Acid and Methyl Jasmonate

(A) Hairy root cultures of *Papaver orientale* were elicited by 100 μM MeJA and 100 μM SA to increase the production of the morphinan alkaloids thebaine, morphine and codeine. MeJA was more effective than SA, resulting in the following improved content: thebaine 3.08 mg/g DW (2.63-fold higher than the untreated control), morphine 5.38 mg/g DW (6.18-fold higher) and codeine 2.57 mg/g DW (3.67-fold higher) [50].

(B) Improved accumulation of terpene indole alkaloids in hairy root cultures of *Rhazya stricta* was obtained by elicitation with MeJA at different concentrations. Treatment with 100 µM MeJA induced a significant increase in the total content of vindoline-type alkaloids [58].

(C) The elicitation of *Taxus x media* var. *Hicksii* hairy roots with 100 μM MeJA proved a highly efficient strategy to enhance taxane production, especially paclitaxel. The production of total paclitaxel (intracellular + released to the medium) increased 3-fold after 7 days of elicitation, reaching a maximum of 1432.5 μg/g DW after 14 days in a perfluorodecalin-supported system [15].

#### 4.1.5. Pectin

Hairy root cultures of *Solanum melongena* were treated with a range of elicitors at different concentrations to increase the content of solasodine, a commercially important alkaloid used as a precursor for the production of complex steroidal compounds. The tested elicitors were YE (1 g/L, 2 g/L, 3 g/L), SA (50, 100, 200, 500 µM) and pectin (0.5%, 1%, 1.5%, 2%). Pectin 1% was found to be the most efficient elicitor to enhance solasodine production, the yield (151.23 μg/g DW) representing a 23-fold increase compared to control hairy roots (6.5 μg/g DW) and up to 88-fold compared to field grown plants (1.71 μg/g DW) [54].

#### 4.1.6. Yeast Extract

(A) Hedayati et al. [44] demonstrated that YE can be used as an effective elicitor to increase tropane alkaloids production in hairy root cultures of *A. belladonna*, although it has a negative effect on growth. Different concentrations of YE (0, 0.5, 1 and 1.5 mg/L) an exposure times (24 and 48 h) were tested. The highest content of scopolamine (9.21%) and atropine (43.39%) were obtained by 1 and 1.5 mg/L YE, respectively, representing a 9- and 5-fold improvement compared to the control.

### 4.2. Phenols

#### 4.2.1. Acetylsalicylic Acid and Salicylic Acid

(A) Rosmarinic acid production in *Prunella vulgaris* L. hairy roots increased 1.3-fold relative to the control hairy roots after elicitation with 6.9 mg/L SA (58.3 mg/g DW) [79].

#### 4.2.2. Chitosan

(A) Jiao et al. [64] found that elicitation of *Isatis tinctoria* L. hairy root cultures by CS was the best treatment to enhance the production of pharmacologically active flavonoids. Compared to control conditions (2.31 ± 0.29 mg/g DW), a 7.08-fold enhancement of total flavonoids (16.35 ± 0.88 mg/g DW) was achieved in 24-day-old *I. tinctoria* hairy root cultures elicited by 150 mg/L CS for 36 h. Interestingly, in these conditions, the significant increase in multiple hydroxyl-substituted flavonoids (rutin, quercetin, isorhamnetin, and isoliquiritigenin) was accompanied by a significant up-regulation of flavonoid biosynthetic genes.

(B) The effect of CS on the production of alizarin, an anticancer phenol produced by *Rubia cordifolia* L. hairy roots, was clearly demonstrated when elicitation with 150 mg/L CS produced a 10-fold higher accumulation compared to control hairy roots after 15 days of cultivation (4.65 ppm versus 0.48 ppm, respectively) [85].

#### 4.2.3. Coronatine

In a study with different tissues of *Linum austriacum*, the effect of elicitation on biomass production and content of justidicin B, a type of lignan synthesized by *Linum* species for plant defense, was tested. Three different in vitro cultures (calli, adventitious roots and hairy roots) were treated with 100 μM MeJA or 10 μM COR. In control samples, the phenol content in hairy roots was double that of calli and adventitious roots. The highest accumulation of total phenols in each tissue was achieved by COR treatment, and the highest overall phenol content was observed in the COR-elicited hairy roots (9.84 μg/mg DW) being 1.5-fold higher than in the control [71].

#### 4.2.4. Jasmonic Acid and Methyl Jasmonate

(A) Daidzin (7-O-glucoside of daidzein) production in hairy roots of *Psoralea corylifolia* L. was enhanced after elicitation with JA. The best response was a 2.8-fold increase in daidzin (5.09% DW) after two weeks of treatment with 1 μM JA and a 7.3-fold increase (3.43% DW) after 10 weeks with 10 μM JA compared to the untreated control. ASA was also assayed, but the results were inferior [86].

(B) *Astragalus membranaceus* hairy root cultures were elicited with MeJA, SA and ASA to increase isoflavonoid production. The optimal enhancement was obtained in hairy roots elicited by 283 μM MeJA. The isoflavonoid yield was 2250.10 ± 71.88 μg/g DW, i.e., a 9.71-fold increase compared to the non-treated control (231.64 ± 6.51 μg/g DW) [69].

(C) Elicitation of *Isatis tinctoria* L. hairy roots with 179.54 mM MeJA increased the content of flavonoids 11.21-fold compared with the control hairy roots. MeJA was more effective than SA and ASA. All three tested elicitors performed best at concentrations of 100–200 mM [43].

(D) *Swertia chirayita* hairy root cultures elicited with 100 μM MeJA was reported as an efficient system for the production of bioactive xanthones. The highest content of swerchirin (0.710 ± 0.13 mg/g DW) and 1,2,5,6-tetrahydroxyxanthone (5.501 ± 0.73 mg/g DW) was obtained only 6 days after elicitation, representing a 1.80- and 6.0-fold increase compared to the control, respectively [83].

(E) Martin et al. [89] reported that the highest production of plumbagin from *Plumbago indica* L. hairy roots was obtained after an elicitation with 50 μM MeJA for 48 h. In these conditions, the yield of plumbagin increased significantly by 5% DW, which was almost 1.5-fold higher compared to the ASA treatment. Additionally, the *Plumbago indica* L. hairy roots were able to produce shoots, and transgenic plantlets were regenerated on MS medium supplemented with 8.8 μM benzyladenine and 2.5 μM indole-3-butyric acid.

(F) Despite reducing growth, elicitation with 100 μM MeJA significantly increased the content of silymarin (1.2 mg/g DW) in *Silybum marianum* hairy roots after 48 h and up-regulated the expression of *lox* (lipoxygenase), *pod* (peroxidase) and *apx* (ascorbate peroxidase) genes [82].

(G) The pharmaceutical potential of *Aster scaber* hairy roots was demonstrated when elicitation with 100 μM MeJA for 4 days dramatically increased the accumulation of total phenolics (244.5 ± 2.5 mg/g gallic acid equivalent (GAE)) and flavonoids (6.7 ± 0.3 mg/g quercetin equivalent (QE)) compared to the non-elicited root cultures (181.65 ± 1.5 and 4.65 ± 0.2 mg/g GAE, mg/g QE respectively). These phenolic compounds included six hydroxycinnamic acids, seven flavonols, seven hydroxybenzoic acids, vanillin, homogentisic acid, and resveratrol [88].

(H) The production of isoflavones by *Glycine max* hairy roots was studied using different concentrations of MeJA and SA. Notably, 72 h exposure to 100 μM MeJA enhanced total isoflavones production in 30-day-old hairy roots (53.16 mg/g DW; 10.67-fold increase), which doubled the production obtained using 200 μM SA at 96 h (28.79 mg/g DW; 5.78-fold increase) [68].

#### 4.2.5. Yeast Extract

(A) The effect of abiotic and biotic elicitors (MeJA, CS, SA, *Agrobacterium* and YE) at various concentrations on total isoflavonoid accumulation was studied in hairy root cultures of *Pueraria candollei*. All elicitors stimulated isoflavonoid production, but 0.5 mg/mL YE was the most efficient resulting in a total isoflavonoid yield (60.5 ± 1 mg/g DW), 4.5-fold higher than in the control [67].

(B) Wilczańska-Barska et al. [84] reported an enhanced secondary metabolite production in *Scutellaria lateriflora* hairy roots after elicitation with 50 mg/L YE. Acetoside increased 1.4-fold (18.5 mg/g DW) and flavone 1.7-fold (14.5 mg/g DW) at 7 and 14 days of elicitation, respectively.

### 4.3. Terpenes

#### 4.3.1. Acetylsalicylic Acid and Salicylic Acid

(A) Hairy roots of *Andrographis paniculate* were elicited with different concentrations of MeJA and SA to increase production of the diterpenoid andrographolide. Both elicitors were most effective at 100 μM, SA produced better results than MeJA, resulting in an eight-fold increase versus a five-fold increase, respectively, compared to the control hairy roots [91]. Adventitious roots of this plant elicited with 25 μM JA increased andrographolide content 4-fold [112].

#### 4.3.2. Chitosan

(A) Hairy root cultures of *Calotropis gigantea* were elicited with MeJA, YE and CS to increase ardenolide production. While all elicitors had a positive effect, the highest cardenolide yield (1.050 mg/L) was obtained with 50 mg/L CS, which was 2.7-fold higher than in the control [94].

(B) When hairy root cultures of *Psammosilene tunicoides* were elicited with 200 mg/L CS, the total triterpenoid saponin accumulation (14.82 mg/g) increased 4.55-fold compared with the control. This treatment also enhanced the release of saponins to the liquid medium, the maximum (38.6%) being observed at day nine of culture [108].

#### 4.3.3. Coronatine

Vaccaro et al. [90] demonstrated that it was possible to significantly increase the amount of bioactive abietane diterpenes in *Salvia sclarea* hairy roots by transcriptional reprogramming induced by COR, and to a lesser extent, MeJA. Both elicitors significantly improved the accumulation of aethiopinone, but prolonged exposure to MeJA inhibited hairy root growth, which in contrast was unaffected by COR. Based on the aethiopinone content and the final hairy root biomass, the optimal system was considered to be COR treatment for 28 days, when the yield was 24-fold higher (up to 105.34 ± 2.30 mg/L) compared to the basal content in untreated hairy roots. MeJA and COR elicitation also enhanced the synthesis of other bioactive abietane–quinone diterpenes.

#### 4.3.4. Jasmonic Acid and Methyl Jasmonate

(A) JA was found to be a very effective elicitor for the enhancement of production, accumulation and secretion of triterpenoids in two lines of *Calendula officinalis* hairy roots. The addition of 100 μM JA increased the accumulation of oleanolic acid saponins in the hairy root tissue up to 20-fold and notably, the secretion of these compounds to the medium up to 113-fold [100].

(B) Diterpenoid production in *Salvia miltiorrhiza* hairy roots increased after elicitation with 100 μM MeJA: 3.9-fold for cryptotanshinone and tanshinone IIA, 3.0-fold for tanshinone I and 1.3-fold for dihydrotanshinone [104].

(C) Hairy roots of *Panax ginseng* elicited with 100 μM MeJA showed an increase in ginsenoside production compared to the control, the levels of the protopanaxadiol group (Rb1, Rb2, Rb3, Rc, and Rd) being much higher than those of the protopanaxatriol group (Rg1, Re, Rf, and Rg2) [96].

(D) 100 μM MeJA was reported to be the most efficient elicitor for improving glycyrrhizin production in *Glycyrrhiza inflata* hairy roots. At day 5 of elicitation, the content of this secondary metabolite increased 5.7-fold (almost 109 μg/g DW) compared to control roots. Other elicitors such as CS did not affect the glycyrrhizin content [98].

#### 4.3.5. Yeast Extract

(A) A study on tanshinones accumulation in *Salvia castanea* Diels f. tomentosa Stib. hairy root cultures elicited with YE (200 μM), MeJA (200 μM) and Ag^+^ (15 μM) showed that all three elicitors enhanced the tanshinone yields (cryptotanshinone, tanshinone I and tanshinone IIA), but the highest content of each one was obtained with the YE [106].

## 5. The Main Biotic Elicitors Used in Adventitious Roots from 2010 to 2022

Table 2 summarizes the data on elicitation in adventitious root cultures from 2010 to 2022.

Several examples are outlined here, grouping different studies according to the type of secondary metabolite. When a biotic elicitor is not mentioned in a group of secondary metabolites, it is due to the fact that no references have been found.

### 5.1. Phenols

#### 5.1.1. Acetylsalicylic Acid and Salicylic Acid

(A) The content of anthraquinone and phenolic compounds in adventitious root cultures of *Rubia tinctorum* L. was improved by the action of SA. The root growth decreased significantly when the SA concentration was increased to 40 µM. The highest content of anthraquinones was obtained with 20 µM SA (31.47 mg/g DW) but the total anthraquinone yield decreased when the concentration increased to 40 µM, probably due to the reduction in root growth rate. In contrast, the total phenolic contents were higher when using 40 µM SA than 20 µM (31.63 and 30.49 mg/g DW, respectively) [115].

(B) The yield of emodin increased 10-fold and chrysophanol 13-fold in adventitious roots of *Aloe vera* due to the effect of SA [113].

#### 5.1.2. Chitosan

(A) Elicitation of adventitious roots of *Morinda citrifolia* with 131.02 µM CS increase in secondary metabolites such as anthraquinones (103.16 mg/g DW), flavonoids (48.57 mg/g DW) and phenolic compounds (75.32 mg/g DW) [22].

(B) Elicitation of *Morinda coreira* Buck adventitious roots with 0.4 mg/mL CS, observed to be the most effective concentration, resulted in a reduction in growth ratio and biomass (fresh and DW) from day two to eight, with an increase in yield of anthraquinones (292.038 mg/g DW) and phenolics (86.8 mg/g DW) until day 4 [114].

#### 5.1.3. Jasmonic Acid and Methyl Jasmonate

(A) The total phenolic compounds and more specifically, the total flavonoids produced by adventitious roots of *Eleutherococcus koreanum* Nakai were analyzed by Lee et al. [118]. The use of 100 mM MeJA increased the production of flavonoids 1.35-fold, and total phenolic compounds was 1.69-fold compared to untreated roots [118].

(B) *Polygonum multiforum* adventitious roots were treated with different elicitors: MeJA, CS, SA, and YE. HPLC analysis of various bioactive compounds revealed significantly higher elicitation efficiency for MeJA than for the other treatments. An approximately 2-fold increase in root dry weight (22.08 mg/g DW) was induced by 50 μM MeJA compared with the control (10.35 mg/g DW) [117].

(C) Adventitious roots of *Fagonia indica* were treated with MeJA to increase the content of useful secondary metabolites. As a result of the treatment, the production of apigenin was increased 1.6-fold compared with the untreated control [116].

### 5.2. Terpenes

#### 5.2.1. Acetylsalicylic Acid and Salicylic Acid

(A) The yield of ginsenosides of *Panax Ginseng* adventitious roots was increased by elicitation with 100 µM SA [122]. However, the maximum productivity (1 mg/g DW) was lower than the highest previously reported by Marsik et al. [120], 3.7 mg/g DW using JA.

#### 5.2.2. Jasmonic Acid and Methyl Jasmonate

(A) Andrographolide is a diterpene lactone whose production in *Andrographis paniculata* adventitious roots increased 10.8-fold after the first week of elicitation with 25 μM JA compared to the control. The other biotic elicitors tested, SA, ASA and methyl salicylic acid, had less effect [112].

(B) Adventitious roots of *Panax ginseng* were elicited with different concentrations of JA: 5, 10 and 50 mg/mL, resulting in a 2.59-, 2.44- and 2.74-fold increase, respectively, in ginsenoside production compared with untreated roots. The maximum yield was achieved by elicitation with 50 mg/mL JA [120].

(C) Adventitious roots of *Tripterygium wilfordii* Hook, f. were treated with MeJA to increase the production of the diterpene triptolide, and the sesquiterpene alkaloids wilforgine and wilforine. Compared to the control, improvements in production were 2.61-fold for triptolide (17.81 mg/g DW) with 50 mM MeJA; 2.63-fold for wilforgine (152.18 mg/g DW) with 100 mM MeJA (57.75 mg/g DW); and 1.821-fold wilforine (10.771 mg/g DW) with 100 mM MeJA. The root cultures were also elicited with SA (50 mM and 100 mM) but no significant increase in the production of the three compounds was observed [124].

(D) Adventitious roots of *Valeriana amurensis* Smir. ex Kom were elicited with MeJA, JA, SA, CS and YE to enhance valtrate production. The most effective treatment was MeJA at 100 mg/mL, which achieved a valtrate content of 10.58 mg/g DW, 3.6-fold higher than the control [123].

(E) *Panax quinquefolium* adventitious roots treated with 5 mg/L MeJA had a 5.24-fold higher ginsenoside content than the control (43.66 mg/g versus 8.32 mg/g, respectively) [121].

#### 5.2.3. Yeast Extract

(A) Tanshinone (cryptotanshinone and tanshinone IIA) production in adventitious root cultures of *Perovskia abrotanoides* Karel was determined after elicitation with 200 μg/mL YE. The treatment had a greater effect on cryptotanshinone than tanshinone IIA, the maximum level (443.62 μg/g DW) being 3.6-fold higher compared to the control, whereas tanshinone IIA increased 1.3-fold [119].

(B) When adventitious roots of *Panax Ginseng* were treated with YE, both growth and production of ginsenosides were lower than in the control [120].

## 6. The Main Combinations of Biotic Elicitors Used from 2010 to 2022

Biotic elicitors are also used in combination with other elicitors, as well as nutrients or precursors, to achieve better results [41]. Although several compounds may be applied together, here we have focused only on combinations of two elicitors, and at least one of them is biotic. Table 3 summarizes the data on the use of combined elicitation in adventitious and hairy root cultures from 2010 to 2022. When a biotic elicitor is not mentioned in a group of secondary metabolites, it is due to the fact that no references have been found.

### 6.1. In Hairy Roots

#### 6.1.1. Alkaloids

The addition of a statistically optimized mixture of elicitors (134.08 μM JA, 108.85 μM MeJA and 3.5 g/L KCl) after 48 h of fed-batch culture of *Catharanthus roseus* hairy roots resulted in a high production of ajmalicine (123.2 ± 8.63 mg/L), a 4-fold improvement compared to bath cultivation alone [126].

#### 6.1.2. Phenols

(A) Krstić-Milošević et al. [73] studied the effect of SA, JA, MeJA, CS and YE elicitors on growth and xanthone accumulation in two hairy root clones of *Gentiana dinarica*. The highest concentrations of all elicitors strongly increased the content of the xanthone aglycone norswertianin, but reduced the production of its glycoside norswertianin-1-O-primeveroside. The most efficient treatment to enhance norswertianin production was a combination of SA (200 μM) and CS (50 mg/L) applied for 7 days, which yielded a 24-fold increase in norswertianin content.

(B) A combination of biotic and abiotic elicitors was found to be the best treatment in order to increase the contents of plumbagin in *Plumbago indica* hairy roots. The use of a yeast carbohydrate fraction, manganese chloride, copper chloride, CS and MeJA not only significantly enhanced (~1.2 to 2 fold) plumbagin production in a shake flask culture compared with the control, but the co-presence of CS and MeJA also promoted plumbagin release into the culture media. The maximum total plumbagin yield (11.96 ± 0.76 mg g/g DW) was obtained after three days of simultaneous exposure to CS (200 mg/L) and MeJA (80 μM). When hairy roots were transferred from shake flasks to a bioreactor culture, a significant increase in fresh root biomass was recorded at day 20, together with a further improvement in total plumbagin production (13.16 ± 1.72 mg g/g DW) [128].

#### 6.1.3. Terpenes

Ultraviolet-B irradiation and 100 mM MeJA were applied separately and in combination in *S. miltiorrhiza* hairy root cultures to increase tanshinone production (cryptotanshinone, tanshinone I and tanshinone IIA). The combined treatment induced the maximum tanshinone production (28.21 mg/L), which was 4.9-fold higher compared to the control. The content of cryptotanshinone and tanshinone I was higher than that of tanshinone IIA [131].

### 6.2. In Adventitious Roots

#### Phenols

(A) The synergic action of SA and L-Phe increased the content of anthraquinones in adventitious root cultures of *Rubia tinctorum* L. and promoted growth. The maximum anthraquinone content achieved was 30.13 mg/g DW. The combined action of 40 µM SA and 100 µM L-Phe also yielded the highest total phenolic content (35.20 mg/g DW) [115].

(B) Adventitious root suspension cultures of *Morinda citrifolia* treated with CS and pectin enhanced anthraquinone production at 98.9 mg/g DW [22].

## 7. Database Charts and Tables

The data below refer only to hairy roots elicited with a single elicitor, as they are far more numerous than for hairy roots treated with more than one elicitor or for adventitious roots.

**Figure 1:**
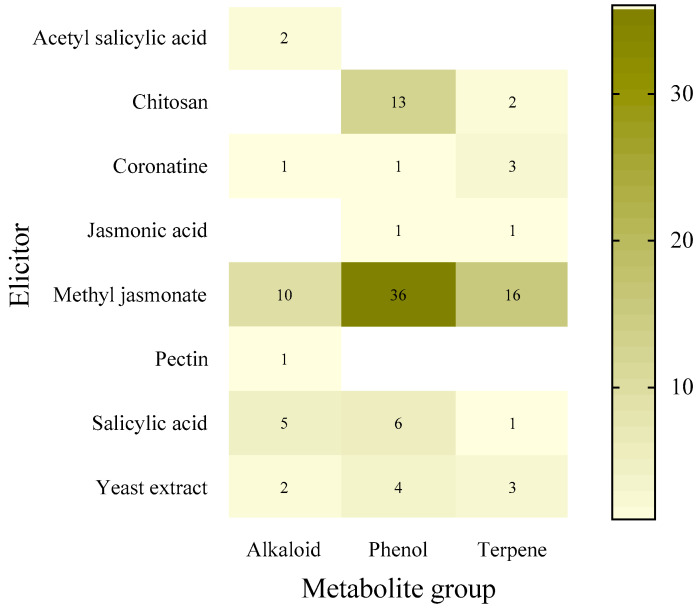
Number of recorded uses of each elicitor in hairy roots by group of secondary metabolites from 2010 to 11 January 2022.

In hairy root cultures, the most frequently used elicitor according to the databases is methyl jasmonate (62 studies), with phenolic compounds being the most studied group of secondary metabolites (61 studies) and alkaloids the least (21 studies) (Figure 1).

**Figure 2:**
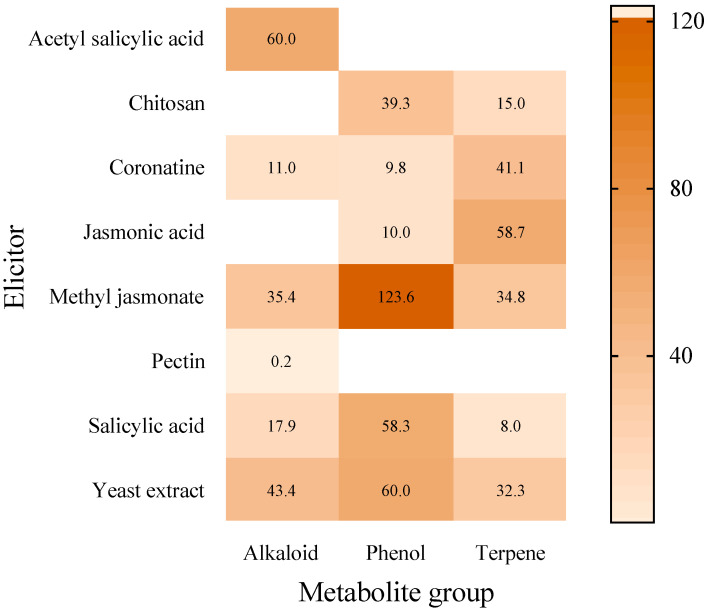
Maximum production (mg/g DW) value of each metabolite group in hairy roots according to the elicitor used, from 2010 to 11 January 2022.

In hairy roots, the highest production value of a phenolic compound was achieved when using MeJA (123.6 mg/g DW) [66], which is the highest value found for all the secondary metabolites. The highest production value for an alkaloid (60 mg/g DW) was obtained by treatment with ASA [56] and for terpenes JA (58.65 mg/g DW) [111] (Figure 2).

In hairy roots, the widest range of elicitor concentration applied was with chitosan (19.7–3650 µM) and yeast extract (0.2–729 µM). The most frequently used elicitor concentration (mode value), regardless of metabolite group, was 100 µM for MeJA and SA. On the other hand, the concentrations of COR are variable according to the metabolite group (Table 4).

**Figure 3:**
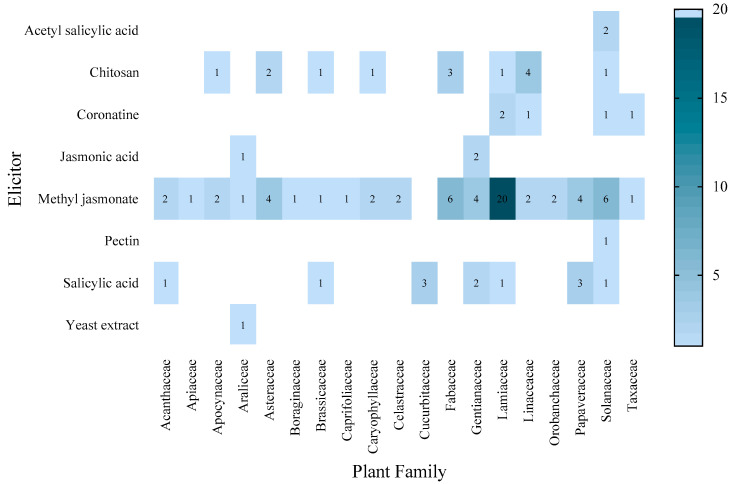
Number of records of each elicitor applied in hairy root cultures by plant family, from 2010 to 11 January 2022.

In hairy root cultures, the Solanaceae are the most studied plant family and have been treated with various elicitors, except JA and YE. The most frequently used elicitor in a particular family is MeJA in the Lamiaceae (20 cases) (Figure 3).

**Figure 4:**
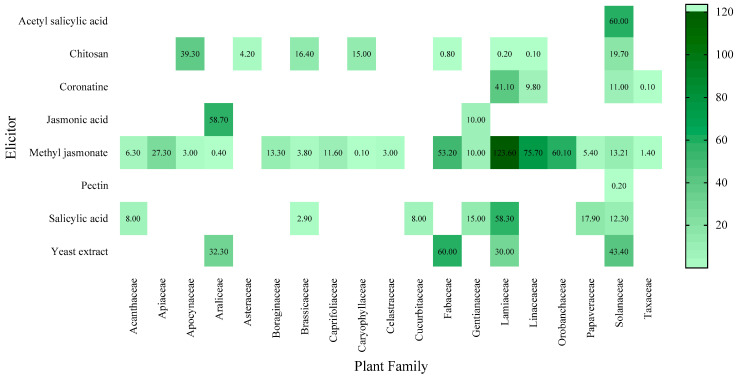
Maximum production (mg/g DW) value achieved by each elicitor according to the plant family, from 2010 to 11 January 2022.

The highest production value of a metabolite (123.6 mg/g DW) found in this work was observed in MeJA-elicited *Salvia virgata*, a member of the Lamiaceae [66] (Figure 4). The maximum production value was reported by a research group from Iran using methyl jasmonate (123.6 mg/g DW) [66] (Figures S1 and S2).

Furthermore, the widest range of elicitor concentration was found for yeast extract applied in the Lamiaceae (0.2–729 µM). The most common value (mode) of elicitor concentration, regardless of plant family, was 100 µM, above all for MeJA and SA (Table 5).

## 8. Concluding Remarks

In the last decade, although the use of in vitro culture of adventitious roots has increased, hairy roots are far more commonly used to obtain PSM. In addition, the production of secondary metabolites after elicitation is always lower in adventitious than in transformed roots, regardless of elicitor and metabolite group. Another interesting fact revealed by this review is that simple elicitation is preferred over combined elicitation, with MeJA being the most used elicitor in both types of cultures. The most frequently targeted secondary metabolites in both types of cultures are phenolic compounds, followed by terpenes and then alkaloids.

In hairy roots, MeJA is the most effective elicitor to increase phenol production, whereas JA gave better results for terpenes, and ASA for alkaloids. In adventitious roots, CS gave the best results for phenol production and MeJA and JA for terpene production. There are no data available for alkaloids in this type of culture.

In hairy roots, the highest values of phenol production (123.6, 80 and 75.65 mg/g DW) were obtained with 100 μM MeJA [66,72,80], respectively. In contrast, in adventitious roots, the highest phenolic production values (292 and 103.16 mg/g DW) were obtained with CS at 262 μM [114] and 131 μM [22], respectively. In relation to terpenes, the highest production was achieved with 20 μM JA (58.65 mg/g DW) [111] and 100 μM JA (52.52 mg/g DW) [100] in hairy roots, and with 22.29 μM MeJA (43.66 mg/g DW) [121] and 25 μM JA (25.48 mg/g DW) in adventitious roots [112].

Regarding alkaloids, the only data found were for hairy roots, 100 μM ASA, 5.46 μM YE and 100 μM MeJA being the best elicitors for production, achieving 60, 43.39, and 35.43 mg/g DW, respectively [44,55,56].

Few data are available for combined elicitation in hairy and adventitious roots (especially the latter). In both types of cultures, combined elicitation has not improved phenolic production. The best phenolic compound yield was achieved in adventitious roots (98.9 mg/g DW) elicited with chitosan (131.02 µM) + pectin (515.09 µM) [22] and in hairy roots (15 mg/g DW) elicited with chitosan (50 mg/L) + SA (200 µm) [73]. Combined elicitation remains more effective than simple elicitation for increasing terpene yield in hairy roots, but not to increase alkaloid production. There are no data for this in adventitious roots. This discussion is summarized in Table 6.

## Figures and Tables

**Table 1:** Elicitation in hairy roots from 2010 to 2022.

Metabolite Group	Plant Species	Plant Family	Secondary Metabolites	Yield/Productivity (mg/g DW)	Elicitor	Concentration (µM)	Reference
**Alkaloid**	*Isatis tinctoria*	Brassicaceae	Alkaloids	2.9	Salicylic acid	100	[43] **
	*Atropa belladonna* L.	Solanaceae	Atropine	43.39	Yeast extract	5.46	[44] *
	*Catharanthus roseus*	Apocynaceae	Catharanthine	0.45	Methyl jasmonate	50	[45]
	*Papaver armeniacum*	Papaveraceae	Codeine	0.12	Methyl jasmonate	100	[46]
	*Atropa belladonna*	Solanaceae	Hyoscyamine	2.1	Methyl jasmonate	40	[47]
	*Datura tatula*	Solanaceae	Hyoscyamine	17.94	Salicylic acid	100	[48] *
	*Datura stramonium*	Solanaceae	Hyoscyamine	12.31	Salicylic acid	100	[49] *
	*Papaver armeniacum*	Papaveraceae	Morphine	0.15	Methyl jasmonate	100	[46]
	*Papaver orientale*	Papaveraceae	Morphine	5.38	Methyl jasmonate	100	[50] *
	*Papaver armeniacum*	Papaveraceae	Noscapine	0.0603	Salicylic acid	100	[46]
	*Papaver armeniacum*	Papaveraceae	Papaverine	0.06	Salicylic acid	100	[46]
	*Echium rauwolfii*	Boraginaceae	Pyrrolizidine alkaloids	13.26	Methyl jasmonate	100	[51]
	*Anisodus luridus*	Solanaceae	Scopolamine	0.068	Acetyl salicylic acid	100	[52] *
	*Atropa belladonna* L.	Solanaceae	Scopolamine	9.21	Yeast extract	5.46	[44] *
	*Solanum trilobatum*	Solanaceae	Solasodine	9.33	Methyl jasmonate	4	[53]
	*Solanum melongena*	Solanaceae	Solasodine	0.15	Pectin	10	[54] *
	*Papaver armeniacum*	Papaveraceae	Thebaine	0.041	Methyl jasmonate	100	[46]
	*Trigonella foenum-graecum*	Fabaceae	Trigonelline	35.43	Methyl jasmonate	100	[55] *
	*Hyoscyamus reticulatus*	Solanaceae	Tropane alkaloids	60	Acetyl salicylic acid	100	[56] *
	*Atropa acuminata*	Solanaceae	Tropane alkaloids	10.95	Coronatine	0.5	[57] *
	*Rhazya stricta*	Apocynaceae	Vindoline-type	0.4	Methyl jasmonate	100	[58] *
**Phenol**	*Mentha spicata*	Lamiaceae	Caffeic acid	0.159	Methyl jasmonate	100	[59]
	*Astragalus membranaceus*	Fabaceae	Calycosin	0.61	Chitosan	65.5	[60]
	*Linum album*	Linaceae	Catechin	0.86	Chitosan	131.01	[61]
	*Lactuca indica* L.	Asteraceae	Chicoric acid	0.06	Methyl jasmonate	50	[62]
	*Lactuca indica* L.	Asteraceae	Chlorogenic acid	0.03	Methyl jasmonate	50	[62]
	*Mentha spicata*	Lamiaceae	Chlorogenic acid	0.015	Methyl jasmonate	100	[59]
	*Mentha spicata*	Lamiaceae	Cinnamic acid	0.043	Methyl jasmonate	100	[59]
	*Lactuca indica* L.	Asteraceae	3,5-dicaffeoylquinic acid	0.12	Methyl jasmonate	50	[62]
	*Ocimum tenuiflorum* L.	Lamiaceae	Eugenol	0.41	Yeast extract	182.28	[63]
	*Isatis tinctoria*	Brassicaceae	Flavonoids	16.35	Chitosan	98.26	[64] *
	*Isatis tinctoria*	Brassicaceae	Flavonoids	3.8	Methyl jasmonate	200	[43] *
	*Momordica charantia* L.	Cucurbitaceae	Flavonols	2.489	Salicylic acid	100	[65]
	*Astragalus membranaceus*	Fabaceae	Formononetin	0.76	Chitosan	65.5	[60]
	*Salvia virgata*	Lamiaceae	Gallic acid	123.6	Methyl jasmonate	100	[66]
	*Momordica charantia* L.	Cucurbitaceae	Hydroxybenzoic acid	7.96	Salicylic acid	100	[65]
	*Momordica charantia* L.	Cucurbitaceae	Hydroxycinnamic acid	1.09	Salicylic acid	100	[65]
	*Pueraria candollei*	Fabaceae	Isoflavanoids	60	Yeast extract	200	[67] *
	*Glycine max*	Fabaceae	Isoflavones	53.16	Methyl jasmonate	100	[68] *
	*Astragalus membranaceus*	Fabaceae	Isoflavonoids	2.25	Methyl jasmonate	283	[69] *
	*Rehmannia glutinosa*	Orobanchaceae	Isoverbacoside	1.77	Methyl jasmonate	200	[70]
	*Linum austriacum*	Linaceae	Justidicin B	9.84	Coronatine	10	[71] *
	*Linum album*	Linaceae	6-methoxypodophyllotoxin	39	Chitosan	131.01	[61]
	*Linum mucronatum*	Linaceae	6-methoxypodophyllotoxin	75.65	Methyl jasmonate	100	[72]
	*Gentiana dinarica*	Gentianaceae	Norswertianin	3	Jasmonic acid	200	[73]
	*Gentiana dinarica*	Gentianaceae	Norswertianin	2.5	Methyl jasmonate	200	[73]
	*Gentiana dinarica*	Gentianaceae	Norswertianin	4.5	Salicylic acid	200	[73]
	*Gentiana dinarica*	Gentianaceae	Norswertianin-1-O-primeverosid	10	Jasmonic acid	200	[73]
	*Gentiana dinarica*	Gentianaceae	Norswertianin-1-O-primeverosid	10	Methyl jasmonate	200	[73]
	*Gentiana dinarica*	Gentianaceae	Norswertianin-1-O-primeverosid	15	Salicylic acid	200	[73]
	*Orthosiphon aristatus*	Lamiaceae	Phenolic acids	17.99	Yeast extract	3.28	[74]
	*Arachis hypogaea*	Fabaceae	Phenolics	0.0108	Methyl jasmonate	100	[75]
	*Linum album*	Linaceae	Podophyllotoxin	0.146	Chitosan	131.01	[61]
	*Linum mucronatum*	Linaceae	Podophyllotoxin	11.37	Methyl jasmonate	100	[72]
	*Salvia virgata*	Lamiaceae	Flavonoids	5.09	Methyl jasmonate	100	[66]
	*Agastache foeniculum*	Lamiaceae	Rosmarinic acid	0.213	Chitosan	65.5	[76]
	*Salvia virgata*	Lamiaceae	Rosmarinic acid	18.45	Methyl jasmonate	100	[66]
	*Mentha spicata*	Lamiaceae	Rosmarinic acid	0.055	Methyl jasmonate	100	[59]
	*Lepechinia caulescens*	Lamiaceae	Rosmarinic acid	41.66	Methyl jasmonate	300	[77]
	*Salvia przewalskii*	Lamiaceae	Rosmarinic acid	65	Methyl jasmonate	400	[78]
	*Prunella vulgaris*	Lamiaceae	Rosmarinic acid	58.3	Salicylic acid	50	[79] *
	*Salvia virgata*	Lamiaceae	Salvianolic acid	2.11	Methyl jasmonate	100	[66]
	*Salvia miltiorrhiza*	Lamiaceae	Salvianolic acid	80	Methyl jasmonate	100	[80]
	*Salvia przewalskii*	Lamiaceae	Salvianolic acid	21.5	Methyl jasmonate	400	[78]
	*Silybum marianum*	Asteraceae	Silymarin	0.705	Chitosan	19.65	[81]
	*Silybum marianum*	Asteraceae	Silymarin	1.2	Methyl jasmonate	100	[82] *
	*Swertia chirayita*	Gentianaceae	Swerchirin	0.71	Methyl jasmonate	100	[83] *
	*Swertia chirayita*	Gentianaceae	1,2,5,6-tetrahydroxyxanthone	5.5	Methyl jasmonate	100	[83] *
	*Rehmannia glutinosa*	Orobanchaceae	Verbacoside	60.07	Methyl jasmonate	200	[70]
	*Linum album*	Linaceae	Vitexin	0.44	Chitosan	131.01	[61]
	*Scutellaria lateriflora*	Lamiaceae	Wogonin	30	Yeast extract	0.18	[84] *
	*Rubia cordifolia* L.	Rubiaceae	Alizarin	4.65 ppm **	Chitosan	98.3	[85] *
	*Psoralea corylifolia*	Fabaceae	Daidzin	0.02% DW **	Jasmonic acid	1	[86] *
	*Solanum trilobatum*	Solanaceae	Flavonoids	521.09 mg/g dry extract (DE) **	Methyl jasmonate	4	[53]
	*Brassica rapa*	Brassicaceae	Glucosinolates	85 µmol/g DW **	Jasmonic acid	50	[87]
	*Aster scaber*	Asteraceae	Phenolic compounds	244 mg/g GAE **	Methyl jasmonate	100	[88] *
	*Solanum trilobatum*	Solanaceae	Phenolics	150.42 mg/g DE **	Methyl jasmonate	4	[53]
	*Plumbago indica*	Plumbaginaceae	Plumbagin	5% DW **	Methyl jasmonate	50	[89] *
**Terpene**	*Salvia sclarea*	Lamiaceae	Abietane diterpenes	41.09	Coronatine	0.1	[90] *
	*Salvia sclarea*	Lamiaceae	Aethiopinone	20.36	Methyl jasmonate	100	[90] *
	*Andrographis paniculata*	Acanthaceae	Andrographolide	6	Methyl jasmonate	100	[91] *
	*Andrographis paniculata*	Acanthaceae	Andrographolide	8	Salicylic acid	100	[91] *
	*Astragalus membranaceus*	Fabaceae	Astragaloside	0.007	Chitosan	3650	[92]
	*Astragalus membranaceus*	Fabaceae	Astragaloside	5.5	Methyl jasmonate	157.4	[93]
	*Taxus media*	Taxaceae	Baccatin III	0.076	Coronatine	1	[15]
	*Calotropis gigantea*	Apocynaceae	Cardenolide	39.3	Chitosan	32.75	[94] *
	*Centella asiatica*	Apiaceae	Centellosides	27.25	Methyl jasmonate	50	[95]
	*Panax ginseng*	Araliaceae	Gingenosides	0.42	Methyl jasmonate	100	[96] *
	*Panax quinquefolium*	Araliaceae	Ginsenosides	32.25	Yeast extract	182.28	[97]
	*Glycyrrhiza inflata*	Fabaceae	Glycyrrhizin	34.79	Methyl jasmonate	100	[98] *
	*Silene linicola*	Caryophyllaceae	20-hydroxyecdysone	0.138	Methyl jasmonate	100	[99]
	*Lepechinia caulescens*	Lamiaceae	Oleanolic acid	0.57	Methyl jasmonate	300	[77]
	*Calendula officinalis*	Asteraceae	Oleanolic acid glycosides	52.52	Jasmonic acid	100	[100] *
	*Taxus media*	Taxaceae	Paclitaxel	1.44	Methyl jasmonate	100	[15]
	*Ajuga bracteosa*	Lamiaceae	Phytoecdysteroids	4.49	Coronatine	1	[101]
	*Rhinacanthus nasutus*	Acanthaceae	Rhinacanthin	6.3	Methyl jasmonate	10	[102]
	*Catharanthus roseus*	Apocynaceae	Tabersonine	3	Methyl jasmonate	250	[103]
	*Salvia przewalskii*	Lamiaceae	Tanshinone II A	0.4	Methyl jasmonate	400	[78]
	*Salvia miltiorrhiza*	Lamiaceae	Tanshinones	2.5	Methyl jasmonate	100	[80]
	*Salvia miltiorrhiza*	Lamiaceae	Tanshinones	0.95	Methyl jasmonate	100	[104] *
	*Salvia miltiorrhiza*	Lamiaceae	Tanshinones	11.33	Methyl jasmonate	100	[105]
	*Salvia castanea*	Lamiaceae	Tanshinones	1.99	Yeast extract	729.12	[106] *
	*Tripterygium wilfordii*	Celastraceae	Triptolide	0.15	Methyl jasmonate	50	[107]
	*Psammosilene tunicoides*	Caryophyllaceae	Triterpenoid saponins	15	Chitosan	131.01	[108] *
	*Silene linicola*	Caryophyllaceae	Turkesterone	0.138	Methyl jasmonate	100	[99]
	*Lepechinia caulescens*	Lamiaceae	Ursolic acid	0.29	Methyl jasmonate	300	[77]
	*Ocimum tenuiflorum* L.	Lamiaceae	Ursolic acid	1.56	Yeast extract	182.28	[63]
	*Valeriana jatamansi*	Caprifoliaceae	Valtrate	11.57	Methyl jasmonate	100	[26]
	*Tripterygium wilfordii*	Celastraceae	Wilforine	3	Methyl jasmonate	50	[107]
	*Withania somnifera*	Solanaceae	Withaferin A	19.65	Chitosan	65.5	[109]
	*Withania somnifera*	Solanaceae	Withaferin A	5.275	Methyl jasmonate	15	[110]
	*Withania somnifera*	Solanaceae	Withanolide A	13.21	Methyl jasmonate	15	[110]
	*Withania somnifera*	Solanaceae	Withanoside IV	0.1929	Methyl jasmonate	15	[110]
	*Withania somnifera*	Solanaceae	Withanoside V	0.161	Methyl jasmonate	15	[110]
	*Panax ginseng*	Araliaceae	Ginsenosides	58.65	Jasmonic acid	20	[111]

* the study is mentioned in the text. ** yield/productivity is represented in other units, DE (dry extract), GAE (gallic acid equivalent).

**Table 2:** Elicitation in adventitious roots from 2010 to 2022.

Metabolite Group	Plant Species	Plant Family	Secondary Metabolites	Yield/Productivity (mg/g DW)	Elicitor	Concentration (µM)	Reference
**Phenol**	*Aloe vera*	Asphodelaceae	Emodin	0.025	Salycilic acid	2000	[113] *
	*Morinda coreia*	Rubiaceae	Anthraquinones	292.038	Chitosan	262.03	[114] *
	*Rubia tinctorum* L.	Rubiaceae	Anthraquinones	31.47	Salycilic acid	20	[115] *
	*Morinda citrifolia*	Rubiaceae	Anthraquinones	103.16	Chitosan	131.02	[22] *
	*Fagonia indica*	Zygophyllaceae	Apigenin	25.3	Methyl jasmonate	2.22	[116] *
	*Polygonum multiflorum*	Polygonaceae	Bioactive compounds	22.08	Methyl jasmonate	50	[117] *
	*Aloe vera*	Asphodelaceae	Chrysophanol	0.55	Salycilic acid	4000	[113] *
	*Morinda citrifolia*	Rubiaceae	Phenolic compounds	75.32	Chitosan	131.02	[22] *
	*Eleutherococcus koreanum*	Araliaceae	Total flavonoids	10	Methyl jasmonate	100	[118] *
	*Morinda coreia*	Rubiaceae	Phenolic compounds	86.8 mg/g GAE **	Chitosan	262.03	[114] *
	*Rubia tinctorum* L.	Rubiaceae	Phenolic compounds	31.63 mg/g GAE **	Salycilic acid	40	[115] *
	*Morinda citrifolia*	Rubiaceae	Phenolics compounds	48.57 mg/g GAE **	Chitosan	131.02	[22] *
	*Eleutherococcus koreanum*	Araliaceae	Total phenolics	22.48 mg/g GAE **	Methyl jasmonate	100	[118] *
**Terpene**	*Andrographis paniculata*	Acanthaceae	Andrographolide	25.48	Jasmonic acid	25	[112] *
	*Perovskia abrotanoides*	Lamiaceae	Cryptotanshinone	0.44	Yeast extract	0.131	[119] *
	*Panax Ginseng*	Araliaceae	Ginsenosides	3.5	Jasmonic acid	23778	[120] *
	*Panax Ginseng*	Araliaceae	Ginsenosides	3.3	Jasmonic acid	47557	[120] *
	*Panax Ginseng*	Araliaceae	Ginsenosides	3.7	Jasmonic acid	237789	[120] *
	*Panax quinquefolium*	Araliaceae	Ginsenosides	43.66	Methyl jasmonate	22.29	[121] *
	*Panax Ginseng*	Araliaceae	Ginsenosides	1	Salycilic acid	100	[122] *
	*Valeriana amurensis*	Caprifoliaceae	Valtrate	10.58	Methyl jasmonate	445.8	[123] *
	*Tripterygium wilfordii*	Celastraceae	Wilforgine	17.81	Methyl jasmonate	50	[124] *
	*Withania somnifera*	Solanaceae	Whitanone	1.13	Salycilic acid	150	[125]
	*Withania somnifera*	Solanaceae	Withaferin a	0.85	Salycilic acid	150	[125]
	*Withania somnifera*	Solanaceae	Withanolide a	1.32	Salycilic acid	150	[125]
	*Withania somnifera*	Solanaceae	Withanolide b	1.16	Salycilic acid	150	[125]

* the study is mentioned in the text. ** yield/productivity is represented in other units, GAE (gallic acid equivalent).

**Table 3:** Combined elicitation in adventitious and hairy roots from 2010 to 2022.

Culture System	Metabolite Group	Plant Species	Plant Family	Secondary Metabolites	Yield/Productivity (mg/g DW)	Elicitor	Reference
**Hairy roots**	Alkaloid	*Catharanthus roseus*	Apocynaceae	Ajmalicine	15.4	Methyl jasmonate (108.85 µM) + Jasmonic acid (134.08 µM) + Potassium chloride (3.5 g/L)	[126] *
	Phenol	*Taxus x media*	Taxaceae	Matairesinol	0.199	Coniferyl alcohol (1 µM) + L-phenylalanine (100 µM) + Methyl jasmonate (100 µM)	[127]
		*Gentiana dinarica*	Gentianaceae	Norswertianin	15	Chitosan (50 mg/L) + Salicylic acid (200 µm)	[73] *
		*Plumbago indica*	Plumbaginaceae	Plumbagin	11.96	Chitosan (200 mg/L) + Methyl jasmonate (80 μM)	[128] *
		*Arachis hypogaea*	Fabaceae	Trans-arachidin-1	684 mg/g DE **	Chitosan (50 mg/L) + Methyl jasmonate (100 µm)+ Cyclodextrin (6.87 mM)	[129]
	Terpene	*Centella asiatica*	Apiaceae	Centellosides	134.6	Coronatine (1 µM) + Methyl jasmonate (100 µM)	[130]
		*Salvia miltiorrhiza*	Lamiaceae	Tanshinones	2.2	Methyl jasmonate (100 µM) + UV	[131] *
		*Salvia miltiorrhiza*	Lamiaceae	Tanshinones	3	β-cyclodextrin + Silver nanoparticles (30 mg/L)	[132]
**Adventitious roots**	Phenol	*Rubia tinctorum*	Rubiaceae	Anthraquinones	30.13	Salycilic acid (20 µM) + L-phenylalanine (50 µM)	[115] *
		*Morinda citrifolia*	Rubiaceae	Anthraquinones	98.9	Chitosan (131.02 µM) + Pectin (515.09 µM)	[22] *
		*Rubia tinctorum*	Rubiaceae	Phenolic compounds	35.2 mg/g GAE **	Salycilic acid (40 µM) + L-phenylalanine (100 µM)	[115] *
		*Arachis hypogaea*	Fabaceae	Trans-arachidin-3	543 mg/g DE **	Chitosan (50 mg/L) + Methyl jasmonate (100 µm)+ Cyclodextrin (6.87 mM)	[129]

* the study is mentioned in the text. ** yield/productivity is represented in other units, DE (dry extract), GAE (gallic acid equivalent).

**Table 4:** Concentration range (µM) of elicitors applied in hairy roots according to the metabolite group, from 2010 to 11 January 2022.

Elicitor		Metabolite Group		
		Alkaloid	Phenol	Terpene
**Acetyl salicylic acid**	Range (µM)	(100–100)	-	-
	Mode (µM)	100	-	-
**Chitosan**	Range (µM)	-	(19.7–3650)	(65.5–131)
	Mode (µM)	-	65.5	ND
**Coronatine**	Range (µM)	(0.5–1)	(10.0–10.0)	(0.1–1)
	Mode (µM)	0.5	10	1
**Jasmonic acid**	Range (µM)	-	(1–200)	(20–20)
	Mode (µM)	-	200	20
**Methyl jasmonate**	Range (µM)	(4–100)	(0.1–400)	(50–400)
	Mode (µM)	100	100	100
**Pectin**	Range (µM)	(10.0–10.0)	-	-
	Mode (µM)	10	-	-
**Salicylic acid**	Range (µM)	(100–100)	(50–200)	(100–1000)
	Mode (µM)	100	100	100
**Yeast extract**	Range (µM)	(5.46–5.46)	(0.2–200)	(182.28–729)
	Mode (µM)	5.46	ND	182.28

**Table 5:** Concentration range and mode value (µM) of each elicitor by plant family, from 2010 to 11 January 2022.

Plant Family	Elicitor															
	Acetyl Salicylic Acid		Chitosan		Coronatine		Jasmonic Acid		Methyl Jasmonate		Pectin		Salicylic Acid		Yeast Extract	
	Range (µM)	Mode (µM)	Range (µM)	Mode (µM)	Range (µM)	Mode (µM)	Range (µM)	Mode (µM)	Range (µM)	Mode (µM)	Range (µM)	Mode (µM)	Range (µM)	Mode (µM)	Range (µM)	Mode (µM)
**Acanthaceae**									(10–10)	10			(100–100)	100		
**Apiaceae**									(50–50)	50						
**Apocynaceae**			(32.8–32.8)	32.8					(50–250)	ND						
**Araliceae**							(20–20)	20	(100–100)	100					(182.3–182.3)	182.3
**Asteraceae**			(19.7–66)	ND					(0.1–100)							
**Brassicaceae**	(50–50)	50	(98.3–98.3)	98.3					(200–200)	200			(100–100)	100		
**Caprifoliaceae**									(100–100)	100						
**Caryophyllaceae**			(131–131)	131					(100–100)	100						
**Celastraceae**									(50–50)	50						
**Cucurbitaceae**													(100–100)	100		
**Fabaceae**			(65.5–3650)	65.5			(1–1)	1	(100–283)	100					(200–200)	200
**Gentianaceae**							(200–200)	200	(0.1–200)	200			(200–200)	200		
**Lamiaceae**			(65.5–65-5)	65.5	(0.1–1)	ND			(100–400)	100			(50–50)	50	(0.2–729)	182.3
**Linaceaeae**			(131–131)	131	(10–10)	10			(100–100)	100						
**Orobanchaceae**									(200–200)	200						
**Papaveraceae**									(100–100)	100			(100–100)	100		
**Solanaceae**	(100–100)	100	(65.5–65.5)	65.5	(0.5–0.5)	0.5			(4–40)	15	(10–10)	10	(100–100)	100	(5.5–5.5)	5.5
**Taxaceae**					(1–1)	1			(100–100)	100						

**Table 6:** Summary of the most frequently used elicitors and those that produce the best results for each group of secondary metabolites (from 2010 to 2022).

	Elicitor	Phenols (Most Studies)	Terpenes	Alkaloids
**Hairy roots** **(most production)**	Most used	MeJA	MeJA	MeJA
	Best for production	MeJA	JA	ASA
**Adventitious roots**	Most used	MeJA	MeJA	-
	Best for production	CS	MeJA/JA	

ASA: Acetylsalicylic acid; COR: Coronatine; CS: Chitosan; JA: Jasmonic acid; MeJA: Methyl jasmonate.

## Data Availability

The new data presented in this study are available within the article.

## Supplementary Materials

The following are available online at https://www.mdpi.com/article/10.3390/molecules27165253/s1, Table S1: Number of records for each elicitor according to the country of origin of the research group, from 2010 to 11 January 2022. Table S2. Number of records of metabolite group studied by research group origin, from 2010 to 11 January 2022. Figure S1. Maximum production (mg/g DW) value obtained in hairy roots according to the country of origin of the research group, from 2010 to 11 January 2022. Figure S2. Maximum production (mg/g DW) value achieved in hairy roots according to the elicitor used and country of origin of the research group, from 2010 to 11 January 2022. Figure S3. Mode concentration (µM) of elicitors used by research groups according to their country of origin, from 2010 to 11 January 2022. Figure S4. Mode concentration (µM) value by elicitor in each research group origin, from 2010 to 11 January 2022. Figure S5. Maximum production (mg/g DW) value obtained for metabolite group by country, from 2010 to 11 January 2022. Figure S6. Mode concentration (µM) value of elicitor by metabolite group in each research group origin, from 2010 to 11 January 2022.
