# Supplementary material for: Biotic Elicitors in Adventitious and Hairy Root Cultures: A Review from 2010 to 2022
Source: Molecules. 2022 Aug 17;27(16):5253. doi: 10.3390/molecules27165253 (PMC9416168; doi:10.3390/molecules27165253)
Supplement: Supplementary file 1 [file molecules-27-05253-s001.zip › molecules-1786249-supplementary.pdf]

Supplementary data

The research groups most active in the elicitation of hairy roots are located in Asia, especially Iran (30 studies) and China (23 studies) (Table S1).

Table S1. Number of records for each elicitor according to the country of origin of the research group, from 2010 to January 11, 2022.

**Research Group Origin**	**Elicitor**							
	Acetyl salicylic acid	Chitosan	Coronatine	Jasmonic acid	Methyl jasmonate	Pectin	Salicylic acid	Yeast extract
**Algeria**							2	
**China**	1	5			14		2	1
**Egypt**					1			
**Germany**								1
**India**		1		1	10	1	1	2
**Iran**	1	6	1		18		2	2
**Iraq**		1						
**Italy**			2		1			
**Mexico**					3			
**Pakistan**			1					
**Poland**			1		3			2
**Republic of Korea**		1		1	4		3	
**Russia**					2			
**Serbia**				2	2		2	
**Thailand**					3			1
**United States**					1			

The maximum production value was reported by a research group from Iran using methyl jasmonate (123.6 mg/g DW) [66] (Figures S1 and S2). Chitosan and pectin have only been used by research groups in Asia. Furthermore, methyl jasmonate was the only elicitor used by A research group in United States of America (Figure S2).

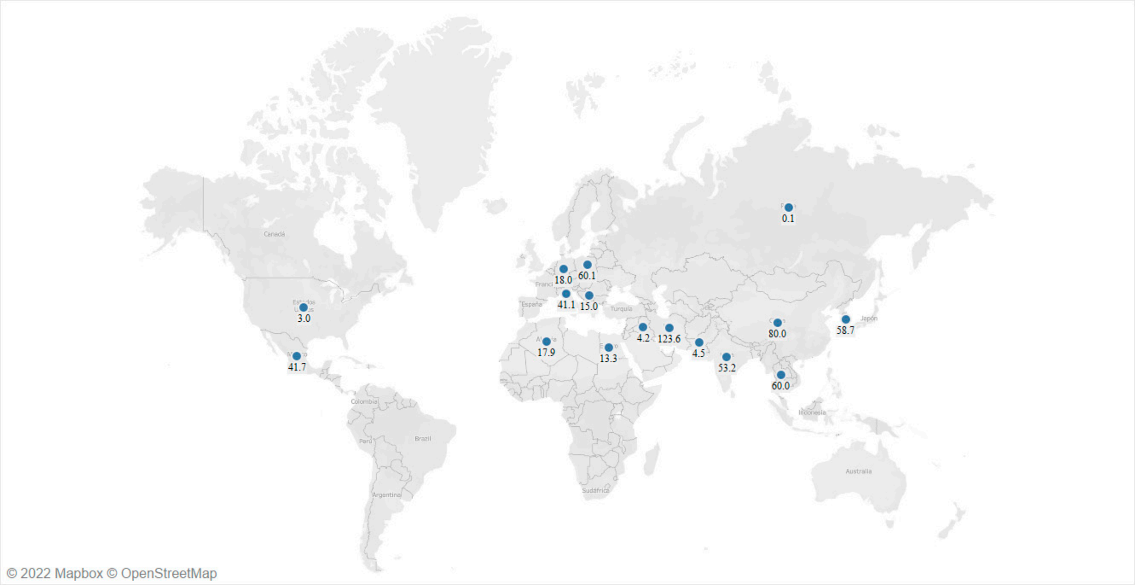

Figure S1. Maximum production (mg/g DW) value obtained in hairy roots according to the country of origin of the research group, from 2010 to January 11, 2022.

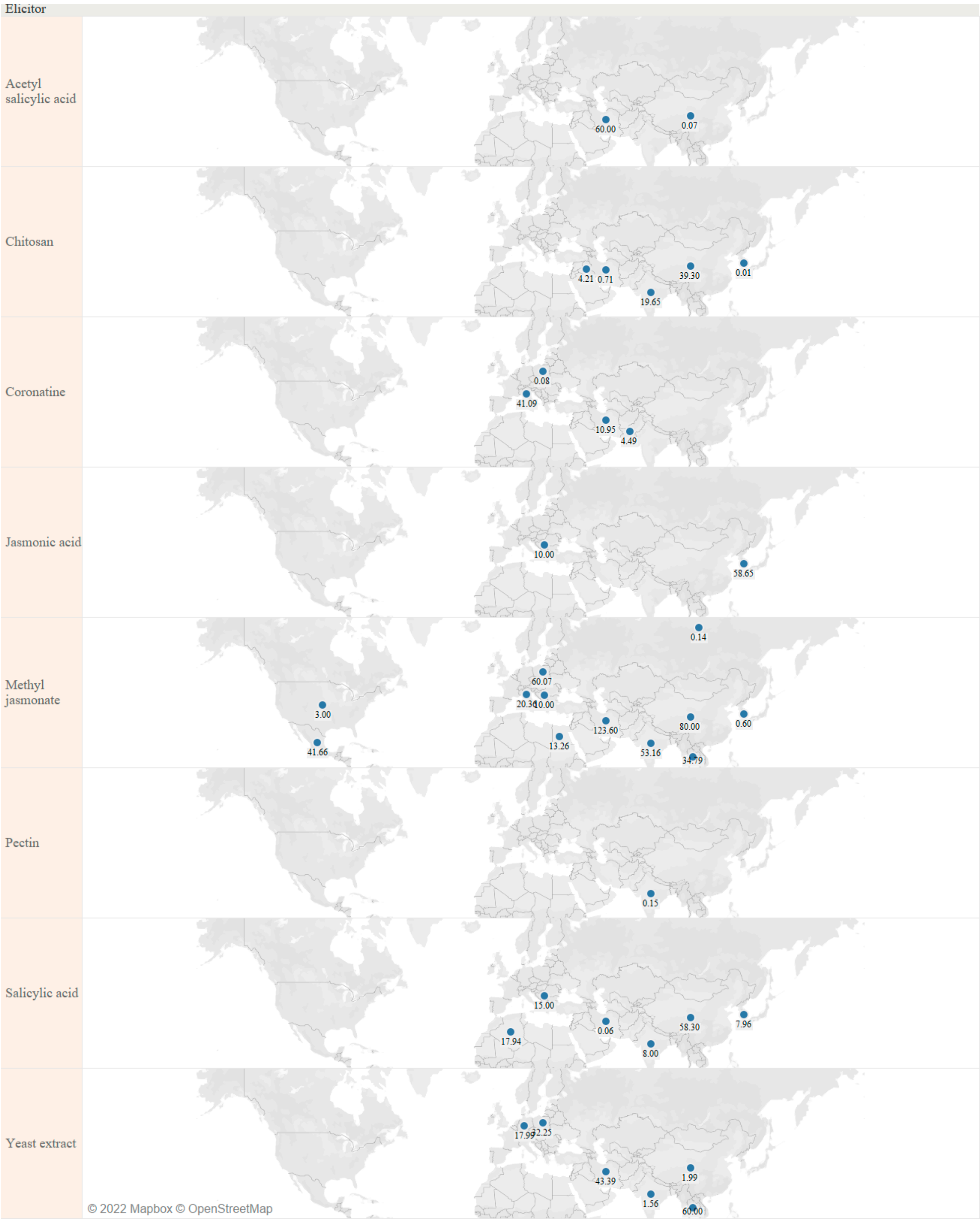

Figure S2. Maximum production (mg/g DW) value achieved in hairy roots according to the elicitor used and country of origin of the research group, from 2010 to January 11, 2022.

The most common value (mode) elicitor concentration used, regardless of the origin of the research group, is 100 µM (Figure S3), especially with MeJA and SA (Figure S4). On

the other hand, the concentration of yeast extract varies according to the country of origin (Figure S4).

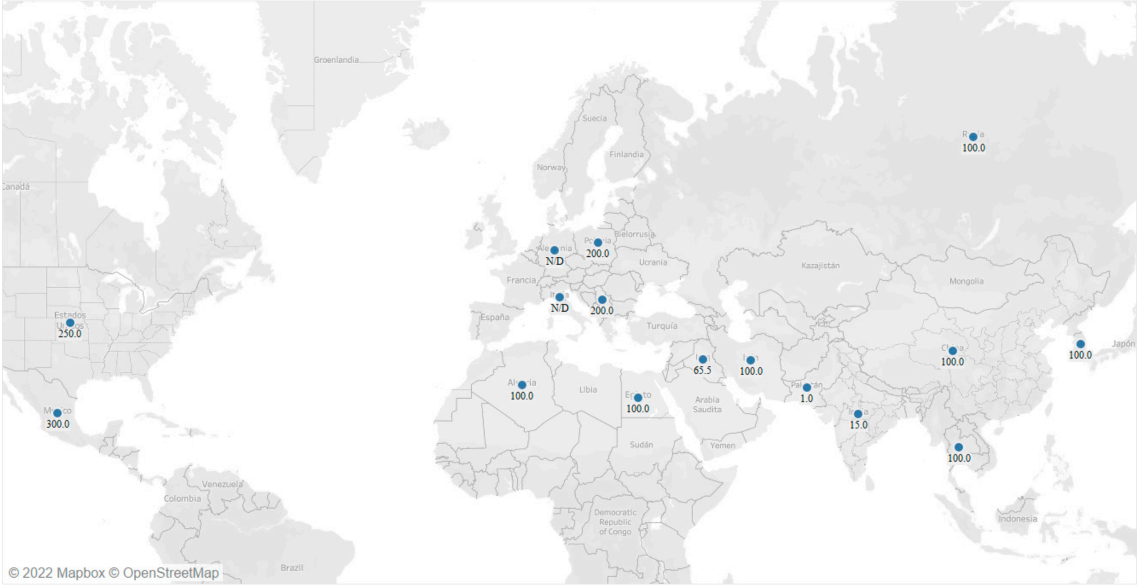

Figure S3. Mode concentration (μM) of elicitors used by research groups according to their country of origin, from 2010 to January 11, 2022.

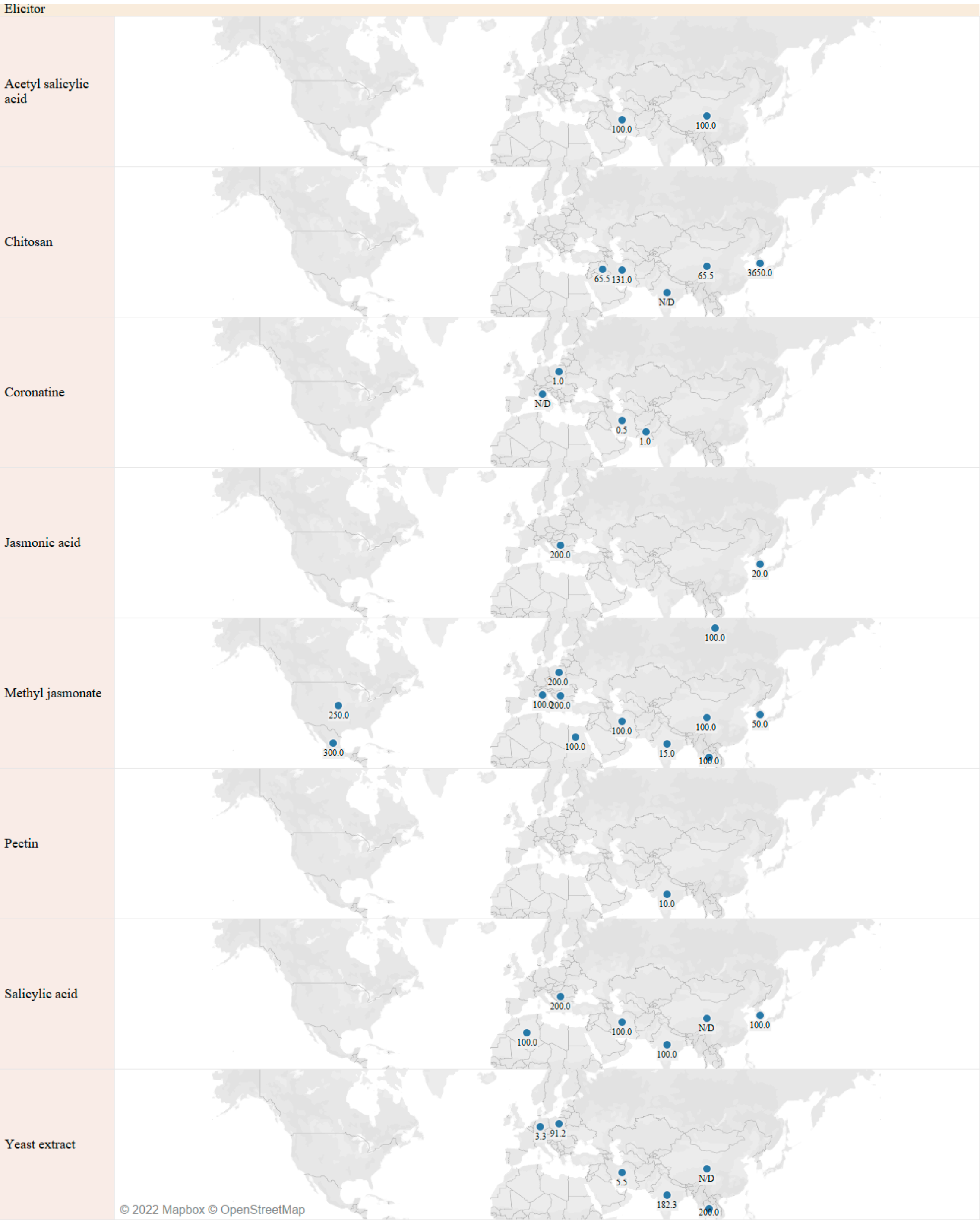

Figure S4. Mode concentration (μM) value by elicitor in each research group origin., from 2010 to January 11, 2022.

Alkaloids and phenolic compounds are the most studied metabolites in Iran (12 and 18 cases, respectively), while terpenes are the most studied in China (9 cases) (Table S2).

The highest production values are mainly for phenolic compounds, especially in Asia, and the maximum value (123.6 mg/g DW) was reported by a group from Iran [66]. The highest production values for alkaloids and terpenes were obtained in Iran and North Korea (60 and 58.65 mg/g DW, respectively) [56,111] (Figure S5).

The most common elicitor concentration is 100 µM, regardless of the origin of the research group. Alkaloids have been studied mainly in Asia and Africa. Phenols and terpenes have been studied by research groups worldwide (Figure S6).

Table S2. Number of records of metabolite group studied by research group origin, from 2010 to January 11, 2022.

**Research Group Origin**	**Metabolite Group**		
	Alkaloid	Phenol	Terpene
**Algeria**	2		
**China**	3	11	9
**Egypt**	1		
**Germany**		2	
**India**	3	14	3
**Iran**	12	18	
**Iraq**			1
**Italy**		1	2
**Mexico**		1	2
**Pakistan**			1
**Poland**		3	3
**Republic of Korea**		8	2
**Russia**		2	
**Serbia**		6	
**Thailand**		2	2
**United States**			1

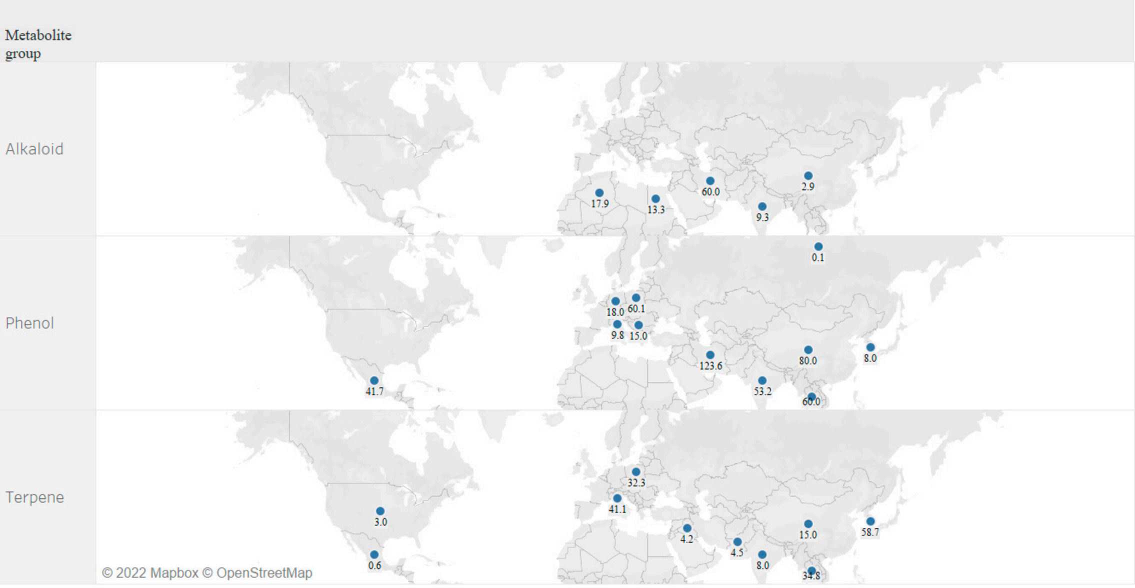

Figure S5. Maximum production (mg/g DW) value obtained for metabolite group by country, from 2010 to January 11, 2022.

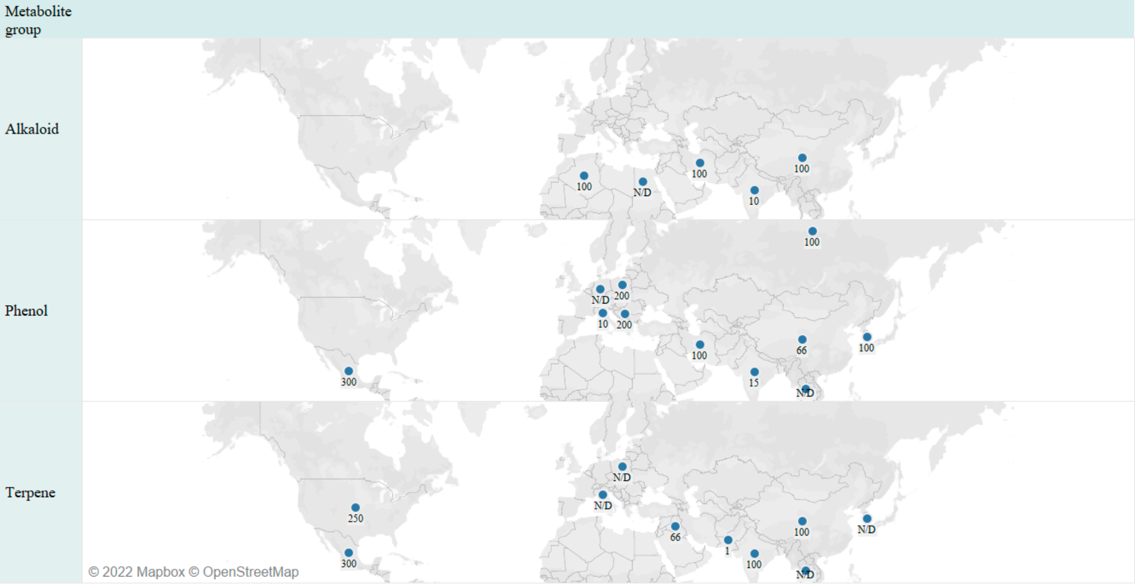

Figure S6. Mode concentration (μM) value of elicitor by metabolite group in each research group origin, from 2010 to January 11, 2022.
